# Seasonal Variability in Marine Atmospheric Mercury Isotope Signatures and Environmental Drivers

**DOI:** 10.1021/acs.est.5c11334

**Published:** 2025-12-22

**Authors:** Zhengcheng Song, Xin Miao, Congyuan Li, Yujuan Wang, Shaojian Huang, Peng Zhang, Kaihui Tang, Tengfei Yuan, Yanxu Zhang

**Affiliations:** † State Key Laboratory of Environmental Geochemistry, Institute of Geochemistry, Chinese Academy of Sciences, Guiyang 550081, China; ‡ School of Atmospheric Sciences, 12581 Nanjing University, Nanjing, Jiangsu 210023, China; § Frontiers Science Center for Critical Earth Material Cycling, Nanjing University, Nanjing, Jiangsu 210023, China; ∥ College of Meteorology and Oceanography, 58294 National University of Defense Technology, Changsha 410073, China; ⊥ University of Chinese Academy of Sciences, Beijing 100049, China; # Department of Earth and Environmental Sciences, 5783 Tulane University, New Orleans, Louisiana 70118, United Sates

**Keywords:** atmospheric mercury isotopes, mass-independent fractionation, marine boundary layer, seasonal variations

## Abstract

Atmospheric deposition is the dominant source of mercury (Hg) to the ocean, shaping its transport, transformation, and potential exposure to global populations. Mercury isotopes offer a powerful tool for evaluating these processes; however, the isotopic composition of atmospheric Hg over the ocean remains poorly understood. This study applies a three-dimensional isotopic model to investigate the mass-independent fractionation (MIF, represented by Δ^199^Hg and Δ^200^Hg) signatures in atmospheric Hg within the marine boundary layer. The model reveals marked spatial and seasonal variations in Δ^199^Hg and Δ^200^Hg of oxidized Hg species. These variations are driven by sea salt aerosol debromination, influenced by environmental factors such as solar radiation, sea surface temperature, and wind speed, which modulate Hg redox chemistry and deposition processes. Simulations indicate that seasonal changes in atmospheric MIF signatures substantially influence the isotopic composition of Hg deposited to the ocean, particularly through dry and wet deposition of oxidized Hg species. These findings underscore the importance of accounting for spatial and temporal variability in atmospheric end-members when tracing marine Hg sources and interpreting sedimentary Hg isotope records under a changing climate.

## Graphical abstract

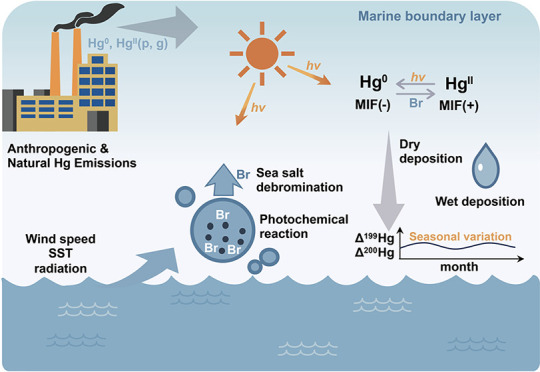

## Introduction

Mercury (Hg) is a globally distributed contaminant with significant adverse health effects on human populations.^1^ Since the Industrial Revolution, the global atmospheric flux of Hg deposition has increased by a factor of approximately 3 to 4 relative to preindustrial levels, primarily due to anthropogenic emissions.^2,3^ The ocean receives Hg mainly through atmospheric deposition, including dry and wet deposition of gaseous oxidized mercury (Hg^II^(g)) and particle-bound mercury (Hg^II^(p)), as well as direct uptake of elemental mercury (Hg^0^) from the atmosphere.^4,5^ In marine environments, inorganic Hg can be converted to monomethylmercury (MeHg), a potent neurotoxin that bioaccumulates and biomagnifies in marine food webs, making seafood consumption the dominant pathway of Hg exposure for humans worldwide.^6,7^ The Minamata Convention on Mercury, a legally binding international treaty aimed at reducing anthropogenic Hg emissions and associated health risks, has been in force since 2017 (https://www.mercuryconvention.org).

Understanding the transport and transformation of Hg in the ocean is critical for protecting human health and evaluating the effectiveness of the Minamata Convention. Over the past two decades, mercury isotope techniques have significantly advanced our understanding of the marine Hg cycle.^8–10^ Mercury has seven stable isotopes that undergo both mass-dependent fractionation (MDF, expressed as δ^202^Hg) and mass-independent fractionation (MIF), which includes odd-MIF (Δ^199^Hg and Δ^201^Hg) and even-MIF (Δ^200^Hg and Δ^204^Hg).^11,12^ MIF signatures are particularly valuable tracers because they are generated only under specific physicochemical conditions. Odd-MIF primarily arises during photochemical transformations, such as the photoreduction of Hg^II^ and the photooxidation of atmospheric Hg^0^.^13–16^ Even-MIF is thought to originate from redox reactions occurring under unique conditions in the upper atmosphere; however, its exact formation mechanism has not been fully evaluated.^16–19^ The distinct three-dimensional isotopic fractionation patterns of Hg provide a powerful tool for tracing its sources and transformation processes in the environment.

Atmospheric deposition is the primary source of Hg to the ocean,^4,5^ yet its isotopic signatures remain poorly characterized, limiting the effectiveness of Hg isotope-based tracing techniques. Current knowledge of Hg isotopes in the marine atmosphere is limited in two key ways. First, insufficient spatial and temporal observational coverage has led to a reliance on terrestrial measurements to infer atmospheric Hg isotopic endmembers for oceanic studies.^20,21^ Second, the isotopic signatures associated with different types of atmospheric deposition—such as dry versus wet deposition or species-specific pathways—remain poorly resolved, despite their critical role in constraining marine Hg sources.^22–24^ These limitations introduce substantial uncertainty into the application of stable Hg isotopes for marine source attribution.

Due to the high cost and logistical challenges of large-scale observational campaigns, conventional techniques are inadequate for capturing the global variability of atmospheric Hg isotopes over the ocean. Recent advances in three-dimensional (3-D) atmospheric Hg modeling provide new opportunities to overcome these challenges.^25,26^ In this study, we employed a global 3-D Hg isotopic model to investigate the spatial and seasonal variability of MIF signatures of atmospheric Hg in the marine boundary layer (MBL). The results reveal distinct MIF patterns among various Hg species, which subsequently influence the distribution and seasonal variability of Hg isotopic signatures in atmospheric deposition to the ocean. Furthermore, we reveal key environmental factors that affect the spatiotemporal distribution of MIF signatures in the MBL and discuss the potential influence on interpreting Hg isotope records in marine sediments.

## Materials and Methods

### Model Description

The three-dimensional mercury isotope model used in this study is based on the GEOS-Chem chemical transport model (version 12.9.0), with a horizontal resolution of 4° latitude × 5° longitude and 47 vertical levels.^4,25^ The model is driven by MERRA-2 meteorological reanalysis data provided by NASA’s Global Modeling and Assimilation Office (GMAO). It incorporates mercury emissions from natural sources—including geogenic, biomass burning, snow-covered land, and oceanic fluxes—as well as anthropogenic sources. Anthropogenic emissions are based on Streets et al.^27^ and further refined by Zhang et al.^28^ The model tracks three atmospheric Hg species with distinct deposition behaviors: Hg^0^, Hg^II^(g), and Hg^II^(p). Hg^0^ undergoes dry deposition exclusively, while Hg^II^(g) and Hg^II^(p) are removed from the atmosphere through both dry and wet deposition processes. The air–sea exchange of Hg^0^ is parametrized using the following set of equations, following the approach of Strode et al.^29^

1 $${\mathrm{Flux}}_{‐\mathrm{up}}=K_{w}\times {\mathrm{CHg}}^{0}$$

2 $${\mathrm{Flux}}_{‐\mathrm{down}}=K_{w}\times {\mathrm{CHg}}_{a}/H$$

The flux of Hg^0^ from the ocean to the atmosphere, denoted as Flux_–up_, is governed by the mass transfer coefficient *K*_w_, which is based on field experiments by Nightingale et al.^30^ CHg^0^ represents the concentration of dissolved Hg^0^ in seawater. CHg^0^ represents the concentration of dissolved Hg^0^ in seawater. Over the ocean, dry deposition primarily occurs in the MBL, where the deposition velocity of Hg^II^(g) is biologically inert and characterized by a high Henry’s law constant.^31^ The dry deposition of Hg^II^(p) is calculated using the aerosol deposition scheme described by Strode et al.^32^ and Zhang et al.^33^ Wet deposition of Hg is linked to precipitation, which scavenges Hg^II^ species from the upper atmosphere, following the framework proposed by Amos et al.^34^

The model utilizes the state-of-the-art chemistry mechanism of atmospheric Hg in GEOS-Chem, which includes the oxidation of Hg^0^ to Hg^I^ by Br, OH and Cl.^4^ The Hg^I^ species can be oxidized to Hg^II^(g) by radicals, and the Hg^II^(g) species can be speciated in aerosol and cloud droplets forming Hg^II^(p). And then both the Hg^II^(g) and Hg^II^(p) species can also be photolyzed back to Hg^I^ and Hg^0^ species. The main chemical processes of atmospheric mercury can be simplified as follows

3 $${\mathrm{Hg}}^{0}+X↔{\mathrm{XHg}}^{I}\left(X=\mathrm{Br},\mathrm{OH and Cl}\right)$$

4 $${\mathrm{XHg}}^{I}+O_{3}\to {\mathrm{XHg}}^{\mathrm{II}}O$$

5 $${\mathrm{XHg}}^{\mathrm{II}}O+\mathrm{CO}\to {\mathrm{XHg}}^{I}+{\mathrm{CO}}_{2}$$

6 $${\mathrm{XHg}}^{I}+{\mathrm{CH}}_{4}\to {\mathrm{XHg}}^{\mathrm{II}}\mathrm{OH}+{\mathrm{CH}}_{3}$$

7 $${\mathrm{XHg}}^{I}+Y+M\to {\mathrm{XHg}}^{\mathrm{II}}Y+M\;\left(Y={\mathrm{HO}}_{2},\mathrm{BrO},\mathrm{ClO}\right)$$

8 $${\mathrm{Hg}}^{\mathrm{II}}\left(p,g\right)+\mathrm{hv}\to {\mathrm{Hg}}^{0}$$

In this study, we focus on simulated Δ^199^Hg and Δ^200^Hg signatures in non-polar marine boundary layer regions, as the mechanisms responsible for odd-MIF in polar regions remain poorly understood and may differ substantially from those in non-polar regions.^35–38^ The MIF signatures assigned to source emissions are summarized in Table S1. The model is initialized with reasonable atmospheric Hg concentrations (Figure S1) and zero MIF signatures. Boundary conditions for land and ocean are also prescribed: the isotopic composition of terrestrial soil Hg is based on organic surface soils reported by Sun et al.^39^ (Δ^199^Hg = −0.25 ± 0.14‰; Δ^200^Hg = 0‰), while the isotopic composition of total Hg in ocean water follows Jiskra et al.^23^ (Δ^199^Hg = 0.08‰; Δ^200^Hg = 0.02‰). The isotopic compositions of major Hg emission sources—including anthropogenic, marine, geological, and biomass combustion—were assumed to be seasonally invariant. In contrast, the odd-MIF signature of soil emissions was modeled as variable with solar radiation, following Zhu et al.^40^ The enrichment factor for terrestrial re-emission of odd-MIF was set to 1.02  ±  0.02‰, consistent with experimental observations of Hg^II^ photoreduction bound to low–molecular-weight thiol (–SH) ligands.^13^ Terrestrial Hg exhibits similar bonding environments, predominantly as thiolate complexes [Hg­(SR)_2_] or Hg–S nanoparticles associated with organic matter in soils and plant leaves.^41,42^ Chemical transformations in the atmosphere can induce MIF in Hg isotopes. All fractionation processes associated with odd-MIF are listed in Table 1, adapted from Song et al.^25^ Specifically, oxidation of Hg^0^ by radicals (Br, Cl, and OH) typically enriches ^199^Hg in the reactant Hg^0^ and depletes it in the products (Hg^I^ and Hg^II^, both gaseous and particulate forms), with enrichment factors of −0.23 ± 0.10‰, −0.37 ± 0.10‰, and −0.18 ± 0.10‰, respectively.^15,43^ The isotopic enrichment factors of oxidation of Hg^I^ to Hg^II^ by O_3_ and BrO are derived from Sun,^43^ who reported fractionation of opposite directions in the Hg^0^ oxidation process: −0.12 ± 0.10‰ for O_3_ and +1.01 ± 0.10‰ for BrO. Isotope fractionation during the photoreduction of Hg^II^(p) process remains less well constrained. As suggested by Sun et al.,^39^ the experiment with a low Hg/DOC ratio (35 ng mg^–1^) is most representative of natural conditions. Therefore, an odd-MIF enrichment factor of −2.75 ± 0.14‰ was applied for atmospheric Hg^II^(p) photoreduction, resulting in enrichment of ^199^Hg in the residual Hg^II^(p) and depletion in the reduced Hg^0^. Comparatively, the odd-MIF associated with photoreduction of Hg^II^(p) is significantly larger than that from Hg^0^ oxidation.

**Table 1:** Hg Isotope Enrichment Factors of Odd-MIF and Even-MIF in Physical and Chemical Processes Contained in the Model^a^

no.	process	*E* ^199^Hg	*E* ^200^Hg	refs
1	Hg^0^ + Br → HgBr^b^	–0.23 ± 0.10‰		^15^
2	Hg^0^ + Cl → HgCl^b^	–0.37 ± 0.10‰		
3	Hg^0^ + OH → HgOH^b^	–0.18 ± 0.10‰		^43^
4	HgY + O3 → Hg^II^(g)^b^ (Y = Cl/Br/OH)	–0.12 ± 0.10‰		
5	HgY + BrO → Hg^II^(g)^b^ (Y = Cl/Br/OH)	1.01 ± 0.10‰		
6	Hg^II^(p)+ *hv*(M) → Hg^0^ ^c^	–2.75 ± 0.14‰	–0.5‰	^13,39,44^
7	Hg^II^OHO + CO → Hg^I^OH^d^		–0.5‰	^44^
7	Hg^0^ emission from soil^e^	eq 1		^40^
8	Hg^0^ re-emission from land^f^	1.02 ± 0.02‰		^39^
9	Hg photoreduction in snow^g^	1.40‰		^25^

a The enrichment factors (*E*) are defined as the ratio of product to reactant.

b Odd-MIF fractionation in oxidation processes is cited from Sun et al.^15^ and Sun et al.^43^

c Enrichment factors for atmospheric Hg^II^ photoreduction process are assumed from Song et al.,^25^ which is the mean enrichment factor of photoreduction for aqueous Hg collected in Sun et al.^39^ Even-MIF in the Hg^II^(p) photoreduction process is speculated in Song et al.^44^

d Even-MIF in the OH-initiated chemical pathway process is speculated in Song et al.,^44^ which is represented by the process of Hg^II^OHO reduction by CO.

e Equation 1 is adopted from Zhu et al.,^40^ the *E* ^199^Hg is a negative correlation with solar radiation (SR, W m^–2^): eq 1: *E* ^199^Hg = −0.0007SR-0.005.

f Photoreduction of terrestrial Hg is utilized from the synthesized data in Sun et al.,^39^ with the *E* ^199^Hg of −1.02‰.

g Chamber measurement^37,38^ has reported *E* ^199^Hg of about 3.5‰ between snow and air during the Arctic AMDE. We speculate that 60% of deposited snowfall-Hg can be re-emission to the air and estimated that the *E* ^199^Hg of 1.40‰ representing the annual level of annual re-emission.

The even-MIF signatures are also generated by chemical processes within the model, although the underlying mechanisms remain incompletely understood. In our recent study,^44^ we systematically evaluated existing hypotheses for even-MIF formation.^16,17,19,45^ Our findings suggest that even-MIF may be primarily driven by OH-initiated redox cycling and Hg^II^(p) photoreduction processes. Based on this interpretation, we developed a “best-case” scenario simulation using an enrichment factor of −0.5‰, which successfully reproduces observed global Δ^200^Hg values across different atmospheric Hg species (Table 1). In this study, seasonal simulations of even-MIF are conducted using the theoretical framework established in our “best-case” scenario. The isotopic compositions of Hg source end-members—including those from natural and re-emission sources—are adopted from Song et al.^25,44^ (Table S1), with natural emissions derived from field observations and anthropogenic emissions based on Sun et al.^46^ We performed model simulations for the years 2016–2018, and the results from the final simulation year (2018) are used for subsequent analysis.

### Synthesized Observation Data

We synthesized observed Δ^199^Hg and Δ^200^Hg data from global atmospheric samples, including gaseous, particulate, and precipitation samples, as shown in Figure 1. Hg^0^ observations are primarily located in East Asia and North America. And Hg^II^(p) data includes observations from both land and marine aerosols. Land observations are mainly from near-shore regions and remote mountain sites. Marine aerosol data were collected from three studies conducted between 2015 and 2019, covering regions from the Southern Ocean to the Arctic Ocean. Huang et al.^47^ conducted three measurement cruises, collecting 52 Hg^II^(p) samples from the western North Pacific, Northwest Pacific, and the northwest marginal region of the Pacific between 2018 and 2019. Qiu et al.^48^ collected and analyzed Hg isotope data from 16 total suspended particle samples in the marginal seas of South and Southeast Asia in 2016. AuYang et al.^49^ reported Hg isotope data from 42 marine aerosol samples collected along a cruise track from Shanghai, China to East Antarctica from 2015 to 2016. The precipitations samples are also primarily located in East Asia and North America. Overall, the data indicate that precipitation samples exhibit the highest levels of odd-MIF and even-MIF values, followed by Hg^II^(p), and then Hg^0^. These data sets include all observed raw data from each site, averaged across the sites, and were used to validate the model’s simulation results.

**Figure 1:**
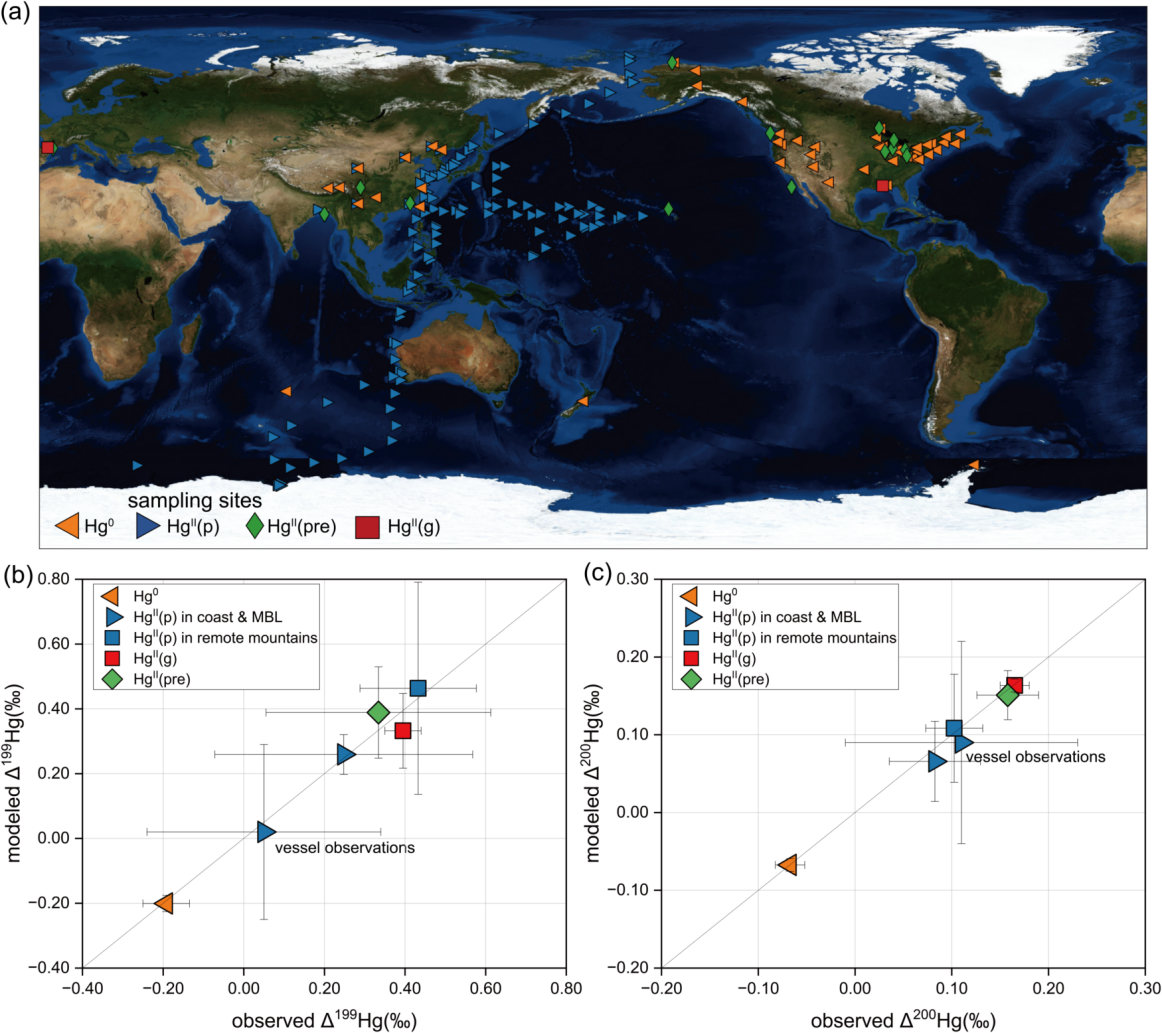
(a) Global map marked with representative observation sites of both odd-MIF and even-MIF signatures in atmospheric Hg from case studies.^18,35,36,38,52–56,59–80^ (b-c) Comparison of observed and modeled mean Δ^199^Hg and Δ^200^Hg signatures for atmospheric Hg^0^, Hg^II^(g), Hg^II^(p), and Hg^II^(pre), respectively. The vessel observations of Hg^II^(p) are cited from three studies covering from Artic Ocean to Antarctic Ocean.^47–49^ The modeled Δ^199^Hg and Δ^200^Hg signatures are annual values calculated from the simulation year.

### Uncertainty

The uncertainties in our model primarily arise from the Hg isotope signatures of source emissions. As shown in Table S1, the Δ^199^Hg and Δ^200^Hg of Hg^0^ emissions has standard deviations ranging from 0.02‰ to 0.18‰ and 0.02‰ to 0.05‰, respectively. Sensitive simulations are conducted to assess the impact of the upper and lower limits (maximum or minimum isotopic ratios) of different emission sources on the modeling results. Results show that the uncertainties of source emissions can lead to max shifts of ± 0.08‰ for modeled Δ^199^Hg^0^ and Δ^199^Hg^II^, and ± 0.03‰ for modeled Δ^200^Hg^0^ and Δ^200^Hg^II^, respectively, which have minimal impact on the analysis of the modeling results.

In addition, the enrichment factors used in the model carry inherent uncertainties (Table 1), which may influence the simulated MIF signatures. To evaluate their impact, we conducted sensitivity simulations by varying the enrichment factors. The results indicate that variations in enrichment factors do not alter the seasonal distribution patterns of the simulated values. As shown in Table S2, doubling the enrichment factor for the oxidation process leads to an increase in the modeled Δ^199^Hg values for Hg^0^ deposition, while Δ^199^Hg values for Hg^II^ dry and wet deposition decrease. However, the magnitude of the seasonal variability, measured as the difference between monthly average maximum and minimum values, remains comparable. Specifically, the variability ranges from 0.02‰ to 0.05‰ for Hg^0^ deposition, approximately 0.1‰ to 0.2‰ for Hg^II^ dry deposition, and about 0.1‰ to 0.4‰ for Hg^II^ wet deposition. Changes in Δ^200^Hg are smaller, as the modeled oxidation process does not induce even-MIF. In contrast, doubling the enrichment factor for the photoreduction of Hg^II^(p) results in an increase in the Δ^199^Hg variability of approximately 0.1‰ to 0.2‰ for dry deposition and 0.2‰ to 0.4‰ for wet deposition. These findings suggest that while uncertainties in enrichment factors influence the magnitude of the modeled MIF values, they do not substantially affect the seasonal trends, supporting the robustness of modeled seasonal patterns.

Another source of uncertainty in the modeled MIF signatures arises from the underlying fractionation mechanism. Although our recent study^44^ demonstrates that the model successfully reproduces most observed Δ^200^Hg values in the global atmosphere, the proposed mechanism remains hypothetical and lacks experimental validation. Furthermore, the model does not account for potential MIF induced by lightning activity, which has been suggested as a contributor to the Δ^199^Hg and Δ^200^Hg signals.^16^ This omission may explain the model’s inability to capture the exceptionally high Δ^200^Hg values (up to 1.24‰) observed in precipitation at Peterborough, Ontario, Canada.^45,50^ In addition, the limited availability of observational data on atmospheric Hg isotopes constrains model validation and introduces uncertainties into the simulation results. In particular, the modeled MIF values and their seasonal variations over the Indian Ocean, South Pacific, and North Atlantic remain unvalidated due to the absence of corresponding observational data, which may contribute to regional uncertainties in the model performance on the MBL environment. Nevertheless, despite these observational limitations, our simulations are based on established Hg isotope fractionation mechanisms, providing confidence in the plausibility of the modeled values for regions where direct measurements are unavailable. Model resolution also introduces uncertainty, particularly in simulating wet deposition. Coarse resolution may underestimate convective wet deposition Hg fluxes,^51^ potentially affecting the modeled isotopic signatures of deposited Hg. Finally, although the model aligns with most available observations, the limited spatial and temporal coverage of observational data may reduce the robustness of model constraints.

## Results and Discussion

### Model Evaluation

The model reproduces the spatial variability of atmospheric Hg concentrations with reasonable accuracy when compared to global observations, as evaluated in our previous studies.^25,44^ Global observations of Hg isotopes, primarily collected from regions distant from major anthropogenic emission sources, are synthesized to validate our model results (Figure 1a). The model successfully reproduces the MIF signatures observed in the global atmosphere. As shown in Figure 1b, the modeled annual mean Δ^199^Hg values for Hg^0^ (−0.20 ± 0.02‰, *n* = 53), Hg^II^(g) (0.33 ± 0.12‰, *n* = 2), Hg^II^(p) in near-shore and MBL environment (0.26 ± 0.06‰, *n* = 5), Hg^II^(p) in remote mountain sites (0.46 ± 0.33‰, *n* = 4), and Hg^II^(pre) (0.39 ± 0.14‰, *n* = 15) are comparable to the observed values of −0.19 ± 0.06‰ (*n* = 53) (*R* ^2^ = 0.15, RMSE = 0.05), 0.40 ± 0.05‰ (*n* = 2), 0.25 ± 0.32‰ (*n* = 5), 0.43 ± 0.14‰ (*n* = 4), and 0.33 ± 0.28‰ (*n* = 15), respectively. This indicates that the odd-MIF simulation method used in this study has good feasibility. Similarly, the modeled annual mean Δ^200^Hg values for various Hg species also align well with observations (Figure 1c). The model results show −0.07 ± 0.01‰ (*n* = 52) for Hg^0^, 0.16 ± 0.01‰ (*n* = 2) for Hg^II^(g), 0.07 ± 0.05‰ (*n* = 4) for Hg^II^(p) in near-shore and MBL environment, 0.11 ± 0.07‰ (*n* = 4) for Hg^II^(p) in remote mountain sites, and 0.15 ± 0.03‰ (*n* = 14) for Hg^II^(pre), which are in close agreement with observed values of −0.06 ± 0.03‰ (*n* = 52) (*R* ^2^ = 0.18, RMSE = 0.02), 0.17 ± 0.02‰ (*n* = 2), 0.08 ± 0.05‰ (*n* = 4) (*R* ^2^ = 0.82, RMSE = 0.02), 0.10 ± 0.03‰ (*n* = 4), and 0.16 ± 0.03‰ (*n* = 14) (*R* ^2^ = 0.50, RMSE = 0.03), respectively. This reflects the feasibility of even-MIF modeling in this study.

We further validated the model results in the MBL by using available marine and coastal atmospheric Hg isotope data. Specifically, when compared with observed Hg isotope compositions of Hg^0^ from island^52–54^ and MBL sites,^55^ the model successfully reproduces the measured Δ^199^Hg (−0.19 ± 0.07‰, *n* = 4) and Δ^200^Hg (−0.06 ± 0.01‰, *n* = 4) signatures, yielding modeled values of −0.18 ± 0.03‰ (*n* = 4) and – 0.06 ± 0.01‰ (*n* = 4), respectively. The model also captures the Δ^199^Hg (0.22 ± 0.1‰) and Δ^200^Hg (0.16 ± 0.05‰) signatures observed at Grand Bay,^56^ which compare favorably with the measurements of 0.35 ± 0.16‰ and 0.18 ± 0.04‰, respectively. In addition, the simulated isotope signatures are consistent with vessel-based observations in the MBL (Figure 1a). These field measurements, collected at hourly to daily resolution,^47–49^ agree well with our annual mean simulation results. Specifically, the observed and modeled Δ^199^Hg values for Hg^II^(p) are 0.05 ± 0.29‰ and 0.02 ± 0.27‰ (*n* = 109), respectively, while the Δ^200^Hg values are 0.11 ± 0.12‰ and 0.09 ± 0.13‰ (*n* = 109), respectively. Moreover, the model reproduces the observed positive latitudinal gradient in both Δ^199^Hg (*r* = 0.54, *P* < 0.05) and Δ^200^Hg (*r* = 0.65, *P* < 0.05) across 60°S to 40°N^48,49^ (Figure S2), indicating that it captures the spatial distribution of Hg^II^(p) MIF values across different marine regions. Overall, the close alignment between the modeled and observed MIF values, which closely approximate a 1:1 relationship (Figures 1b,c), highlights the robust performance of our Hg isotope model.

We also evaluated the modeled seasonal variations of MIF signatures using available observational data. As shown in Figure S3, the simulations generally capture the observed seasonality of Δ^199^Hg and Δ^200^Hg. Specifically, the modeled Δ^199^Hg^0^ and Δ^200^Hg^0^ show positive correlation with observations at remote mountain sites in Changbai and Ailao, China (Figure S3a–d), with correlation coefficient (*r*) ranging from 0.58 to 0.97. The model also reproduces the seasonal variations in Δ^199^Hg^II^(p) and Δ^200^Hg^II^(p) reported at Ailao (*r* = 0.72 and 0.92, respectively; Figure S3e,f) and Changbai (*r* = 0.44 and 0.92, respectively; Figure S3g,h). Nonetheless, some discrepancies remain between simulations and observations. For instance, at mountain Ailao, the modeled Δ^199^Hg of Hg^0^ closely matches seasonal trend but slightly underestimates values by ∼0.1‰. At mountain Changbai, the modeled Δ^199^Hg^II^(p) and Δ^200^Hg^II^(p) are also underestimated. These biases may result from the omission of vegetation re-emission processes in the model, as previous studies have shown that Hg release from foliage can potentially enrich atmospheric Hg in Δ^199^Hg.^57,58^ In contrast, at mountain Waliguan (Figure S3i,j), the model overestimates both Δ^199^Hg^II^(p) and Δ^200^Hg^II^(p). This overestimation likely arises from two sources: (1) uncertainties in the Hg^II^(p) photoreduction enrichment factor, as discussed in the Uncertainty Section, and (2) excessive downdrafts in the model that transport Hg with higher MIF signatures from the upper to lower atmosphere (Figure S4). Differences in meteorological conditions between simulation (2018) and observation years (2014–2015)^59^ may further contribute to these discrepancies. Few studies have reported seasonal MIF variations in precipitation. Compared with observations from Peterborough, Ontario, Canada, the model did not reproduce the seasonal pattern of Δ^199^Hg^II^(pre) but showed a comparable trend for Δ^200^Hg^II^(pre) (Figure S3k,l). These discrepancies likely stem from limitations in model resolution and incomplete representation of MIF-inducing mechanisms (such as potentially lightning-induced fractionations), as discussed in the Uncertainty section. Despite these limitations, the model demonstrates skill in simulating the global distribution and seasonal variability of atmospheric Hg MIF. Notably, in the marine boundary layer—where seasonal observational data are lacking—the model could offer new insights into the spatial and seasonal dynamics of atmospheric mercury isotopic signatures.

### Seasonal Variation of MIF Signatures in MBL

Based on the successful reproduction of global observed MIF signatures, the model reveals distinct seasonal variations in MIF signatures within the MBL. Significant seasonal variations are shown in both Δ^199^Hg and Δ^200^Hg signatures for atmospheric Hg^II^(g) and Hg^II^(p), but not for Hg^0^ (Figure S5). This pattern indicates that chemical fractionation processes primarily drive the modeled seasonality in MIF, while the isotopic composition of Hg^0^ remains stable due to its dominance in the atmospheric Hg pool (>90%) and the resulting reservoir effect, which effectively dilutes chemical fractionation signals. Consequently, we focus below on the seasonal variations of MIF signatures for Hg^II^ species. In general, the MIF signatures exhibit distinct trends across hemispheres (Figure 2). In the Southern Hemisphere MBL, both Δ^199^Hg^II^(p) and Δ^200^Hg^II^(p) values are lower from March to August compared to September to February. In contrast, the MIF values for these same species in the Northern Hemisphere MBL show the opposite seasonal trend (Figure 2).

**Figure 2:**
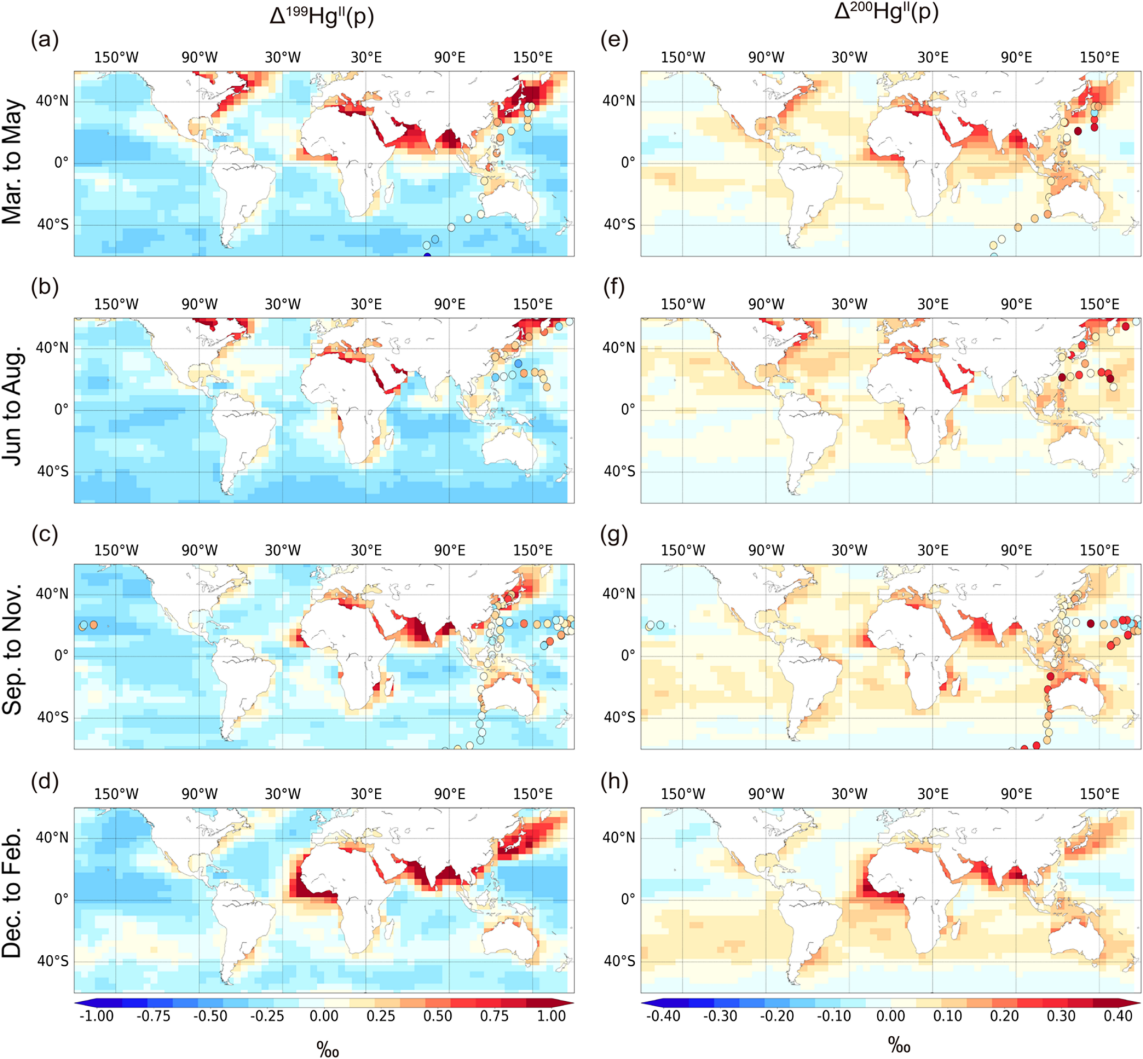
Seasonal variation of MIF signatures of Hg^II^(p) in the MBL of non-polar regions (60°S to 60°N). The seasons is distinguished according to North Hemisphere, which comprises of spring (March to May), summer (Jun to August), autumn (September to November), winter (December to February). (a)–(d) Δ^199^Hg^II^(p) signatures in the four seasons, respectively. (e)–(h) Δ^200^Hg^II^(p) signatures in the four seasons, respectively. Observations are represented points, which are collected from three studies.^47–49^ .

The Δ^199^Hg and Δ^200^Hg values of Hg^II^(g) exhibit a distribution pattern similar to that of Hg^II^(p) and show comparable seasonal variations in the MBL (Figure S6). This phenomenon is attributed to the dynamic exchange between Hg^II^(g) and Hg^II^(p),^4^ resulting in similar MIF signatures for both species. This helps explain why observed Δ^199^Hg and Δ^200^Hg values for Hg^II^(g) in Grand Bay, USA^56^ and Pic du Midi, France^18^ are comparable to those found in Hg^II^(p). The seasonal variations in Δ^199^Hg and Δ^200^Hg values in precipitation are similar to those in Hg^II^ species (Figure S7), as Hg in wet deposition is scavenged from atmospheric Hg^II^(g) and Hg^II^(p).^4,5^ Additionally, precipitation typically originates from the upper atmosphere, where MIF signatures are more pronounced due to intense redox reactions (see Method and Figure S8) and potential aqueous Hg^II^ photoreduction.^14,18,81^ As a result, precipitation tends to have higher MIF values than Hg^II^ species in the MBL.

Near-shore regions generally exhibit higher MIF values compared to the open ocean (Figure 2). This pattern arises from differences in chemical fractionation processes, as redox conditions vary between land surfaces and the MBL: land regions are typically characterized by net reduction, while the MBL tends to exhibit net oxidation (Figure S9). In the model, oxidation of Hg^0^ depletes odd Hg isotopes in the resulting Hg^II^, whereas photoreduction of Hg^II^(p) enriches both odd and even isotopes in the residual Hg^II^(p) (see Methods for details). Consequently, atmospheric Hg^II^ species in the MBL generally display negative Δ^199^Hg and Δ^200^Hg values, while Hg^II^ over land exhibits positive values. These isotopically enriched Hg^II^ species can be transported from terrestrial regions into the MBL, particularly affecting the Δ^199^Hg and Δ^200^Hg signatures in near-shore environments.

To investigate the drivers of seasonal variations in atmospheric Hg^II^ MIF signatures, four representative ocean regions were selected: the North Pacific, North Atlantic, Indian Ocean, and South Pacific (Figure 3a,b). In the MBL of the North Pacific and North Atlantic, Δ^199^Hg^II^(p) and Δ^200^Hg^II^(p) values increase gradually from February–March, peak between July and September, and decline to their minima in December–January (Figures 3c,d–h). In contrast, the Indian Ocean and South Pacific display an opposite seasonal pattern, with MIF values peaking from December to March. These seasonal variations are closely associated with large-scale environmental changes that modulate the oxidative capacity of the MBL. Specifically, the modeled Br concentrations, which are largely controlled by sea-salt aerosol abundance and photochemical activation, exhibit seasonal patterns consistent with the observed MIF variations (Figure 3). Regions with higher Br concentrations—often occurring during periods of strong wind and enhanced sea-salt aerosol production—experience more intense oxidation, resulting in lower Δ^199^Hg^II^(p) and Δ^200^Hg^II^(p) values. Conversely, regions and seasons with reduced Br levels, typically associated with wind, temperature, and sea-salt loading, show elevated MIF values (Figure S10).

**Figure 3:**
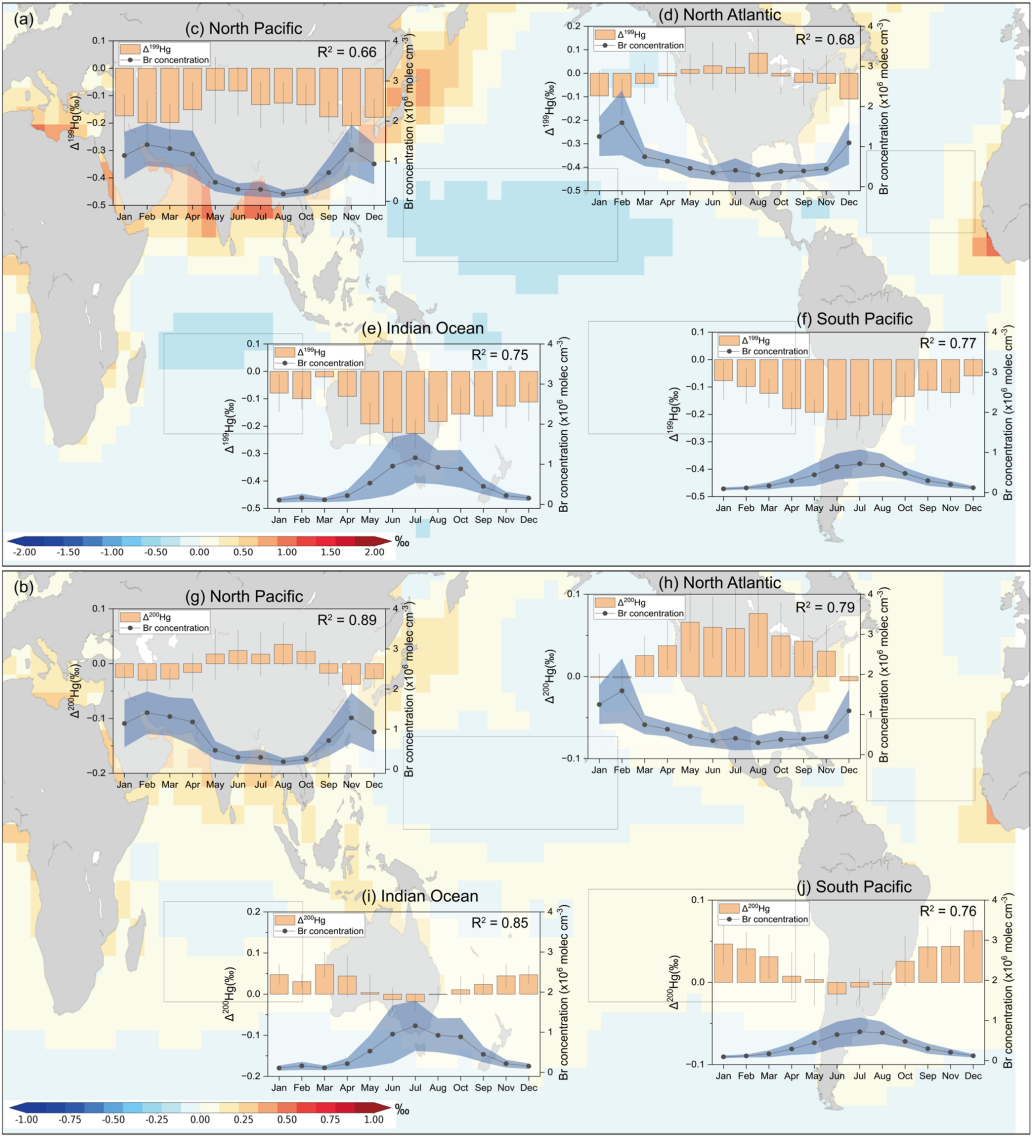
Modeled monthly variations of MIF signatures in the four selected ocean regions. (a) The background color represents modeled annual mean of Δ^199^Hg^II^(p) signatures. (b) The background color represents modeled annual mean of Δ^200^Hg^II^(p) signatures. (c)–(f) Comparison of modeled seasonal Δ^199^Hg^II^(p) and Br concentrations in North Pacific (selected area: 10 ∼ 40°N, 135 ∼ 180°W), North Atlantic (selected area: 10 ∼ 40°N, 30 ∼ 60°W), Indian Ocean (selected area: 0 ∼ 30°S, 60 ∼ 95°E) and Southern Pacific (selected area: 10 ∼ 40°S, 90 ∼ 180°W), respectively. (g)–(i) Comparison of modeled seasonal Δ^200^Hg^II^(p) and Br concentrations in North Pacific, North Atlantic, Indian Ocean and Southern Ocean, respectively.

Further analysis reveals that variations in sea salt aerosol and incident shortwave radiation are the primary drivers of seasonal changes in the MIF signatures of Hg^II^(p) (Figure 4). The modeled Δ^199^Hg^II^(p) and Δ^200^Hg^II^(p) values in the four selected ocean regions show significant correlations with sea salt aerosol abondance, Hg^II^(p) concentrations, net reduction rates, and incident shortwave radiation. In the MBL, sea-salt aerosols, formed under higher wind speed and sea surface temperature,^82–85^ act as both sources of reactive bromine,^86^ and carriers for Hg^II^(g), facilitating the formation of particulate Hg^II^(p).^87^ Moreover, incident shortwave radiation, modulated by seasonal changes in solar angle and cloud cover, directly controls photoreduction rates of Hg^II^(p), which in turn affect its MIF signatures. Taken together, our results indicate that solar radiation, wind speed, and SST are the fundamental environmental drivers regulating the seasonal variability of MIF signatures in atmospheric Hg^II^ species in the MBL. These physical drivers influence the underlying photochemical and redox processes represented by model parameters such as Br concentration and photoreduction rates.

**Figure 4:**
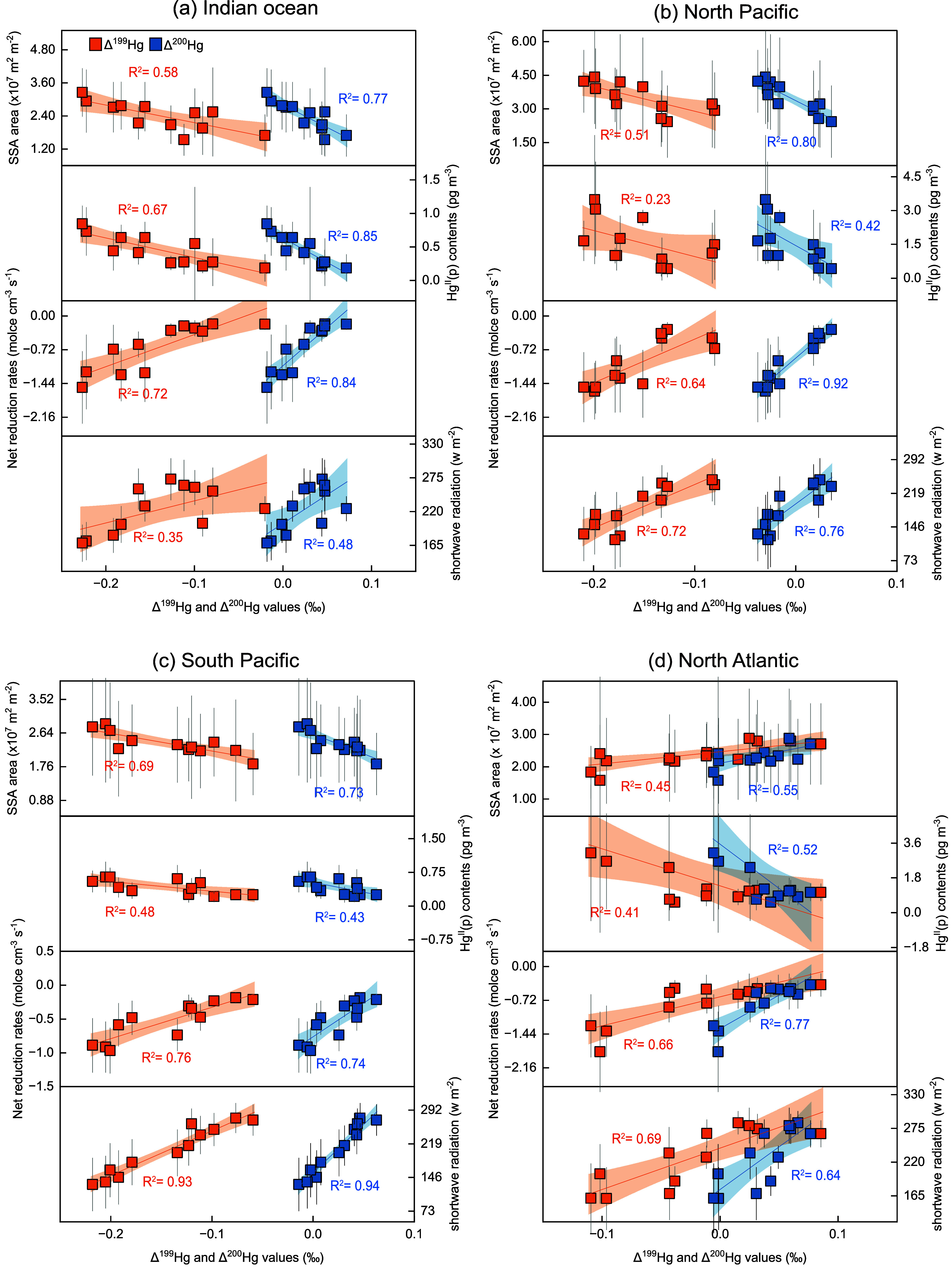
Association between monthly mean values of important factors and MIF signals of atmospheric Hg over: (a) Indian ocean, (b) North Pacific Ocean, (c) South Pacific Ocean, and (d) North Pacific Ocean. The *R* ^2^ values represent correlation coefficients between MIF and corresponding factors, with yellow R^2^ of Δ^199^Hg^II^(p) and blue of Δ^200^Hg^II^(p) values.

### Impact on Deposited Mercury to Ocean

The monthly variation in MIF signatures of atmospheric Hg significantly influences the MIF characteristics of Hg deposited to the ocean. As shown in Figure 5, the model reveals the seasonal dynamics of Δ^199^Hg and Δ^200^Hg for different deposition pathways across the four selected ocean regions. Monthly trends in MIF values vary between the Northern and Southern Hemispheres, consistent with the hemispheric differences in atmospheric Hg isotope signatures discussed above. The MIF signatures also differ by deposition type. Hg^0^ deposition (i.e., oceanic uptake of Hg^0^) exhibits the lowest Δ^199^Hg and Δ^200^Hg values, followed by Hg^II^ dry deposition, while Hg^II^ wet deposition shows the highest MIF values. These differences reflect chemical fractionation processes in the atmosphere, wherein oxidation enriches odd and even isotopes in Hg^II^ and depletes them in Hg^0^. Because the atmospheric Hg^0^ reservoir is significantly larger than that of Hg^II^,^4^ the seasonal variations in Hg^0^ MIF values are much smaller than those observed for Hg^II^. Dry and wet Hg^II^ deposition show broadly similar seasonal trends in MIF values, as both Hg^II^(g) and Hg^II^(p) are the dominant species scavenged during precipitation events. However, because wet deposition originates primarily from the upper atmosphere—where stronger redox activity enhances MIF signatures—the seasonal variations of Δ^199^Hg and Δ^200^Hg in wet deposition is greater than in dry deposition.

**Figure 5:**
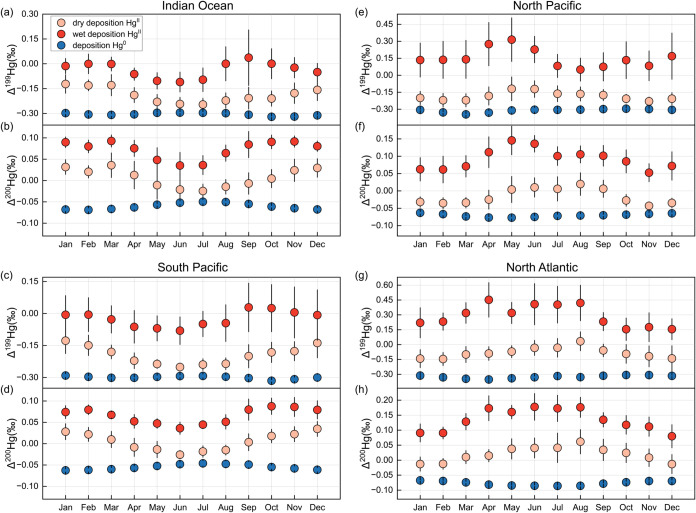
Modeled MIF signatures of various deposited mercury from atmosphere to the four oceans regions. The blue points represent Δ^199^Hg and Δ^200^Hg signatures of Hg^0^ uptake by the ocean regions. The pink and red points represent Δ^199^Hg and Δ^200^Hg signatures of dry deposition Hg^II^ and wet deposition Hg^II^ from atmosphere to the ocean regions, respectively.

### Implications

This study has important implications for the application of Hg isotope techniques in marine environmental research. Given the high cost and logistical challenges associated with measuring atmospheric Hg isotopes over the oceans, our model-based results offer valuable predictive insights to guide and optimize future measurement strategies. It should be emphasized, however, that these conclusions are derived from simulations constrained by limited observational data—particularly over remote oceanic regions such as the central Pacific and the Southern Ocean—thus restricting the direct extrapolation of modeled patterns to these areas. Furthermore, the model does not incorporate the recently proposed lightning-induced MIF mechanism,^16^ which may introduce additional uncertainties in high-latitude regions where this process could be significant.

The model predicts distinct spatial patterns of atmospheric Hg MIF over the ocean, indicating that regional isotopic signatures must be considered when apportioning sources using MIF. Neglecting this spatial variability could significantly compromise source attribution analysis, particularly regarding MIF differences between near-shore and open ocean environments. The model also reveals pronounced seasonal trends and variations among different atmospheric Hg species, underscoring the urgent need to develop isotopic measurement techniques for specific Hg forms—particularly gaseous oxidized Hg and dry deposition. Such advancements are essential for improving our understanding of the transport and transformation of atmospheric Hg in marine environments. Particularly, our findings suggest that future observational efforts should prioritize regions where model–data discrepancies and uncertainties remain high, such as the Indian Ocean, central-eastern Pacific, and the Southern Ocean. Given the substantial contribution of atmospheric Hg^II^ to oceanic mercury inputs,^23,44^ the seasonal variability in the MIF signatures of deposited Hg^II^ may potentially influence the isotopic composition of marine Hg. Coordinated measurements of Δ^199^Hg and Δ^200^Hg in both the atmosphere and surface ocean in these data-scarce areas would be especially valuable for validating model predictions and refining source apportionment frameworks. For instance, our model results indicate a maximum monthly mean difference of 0.30‰ for Δ^199^Hg and 0.10‰ for Δ^200^Hg in dry and wet Hg^II^ deposition in the North Atlantic—an error margin that could meaningfully affect source apportionment and flux estimates.

Furthermore, given that oceanic biological productivity also exhibits strong seasonal cycles,^88^ seasonal fluctuations in atmospheric Hg isotope signatures are likely to propagate through the marine system, influencing the preservation and interpretation of Hg isotope signals in ocean reservoirs. As atmospheric Hg MIF signatures are influenced by environmental factors such as wind speed and solar radiation, the climate variability may alter the isotopic composition deposited to the ocean. Therefore, processes using isotopic records must account for the influence of climatic and oceanographic conditions. Finally, these same environmental drivers significantly affect the transport and deposition of Hg within the marine boundary layer, and are likely to influence Hg exchange at the ocean–atmosphere interface under future climate change scenarios. We recommend that future measurements of Δ^199^Hg and Δ^200^Hg in both the marine boundary layer and ocean interior be interpreted within a seasonal framework to more accurately characterize Hg cycling and transformation processes across the sea–air interface.

## Supplementary Material







## Data Availability

All data generated in this study are available in the main text, the Supporting Information, and the research group Web site: https://www.ebmg.online/mercury.

## Notes

Atmospheric Hg concentrations in the surface air under the initial atmospheric conditions (Figure S1); comparison of observed and modeled latitudinal MIF signatures in Hg^II^(p) (Figure S2); comparison of observed and modeled seasonal variation of MIF signatures of atmospheric Hg (Figure S3); vertical profiles (Figure S4); standard deviations (Δ) of monthly MIF signatures in the marine boundary layer (Figure S5); seasonal variation of MIF signatures of Hg^II^(g) in the MBL of non-polar regions (Figure S6); seasonal variation of MIF signatures of Hg^II^(pre) in the MBL of non-polar regions (Figure S7); annual zonal mean (Figure S8); annual mean chemistry rates (Figure S9); monthly mean of Br contents in global marine boundary layer (Figure S10); isotope signatures of Hg end-members emitted from emission sources (Table S1); min and max monthly mean MIF signatures modeled by sensitive simulations (Table S2) (PDF)
Relevant data generated by the model are presented (XLSX)
All the collected data for validating the modeling results (XLSX)
