# Supplementary material for: Seasonal Variability in Marine Atmospheric Mercury Isotope Signatures and Environmental Drivers
Source: Environ Sci Technol. 2025 Dec 22;60(1):664–76. doi: 10.1021/acs.est.5c11334 (PMC12810244; doi:10.1021/acs.est.5c11334)
Supplement: Supplementary file 1 [file es5c11334_si_001.pdf]

Supporting Information for:

Seasonal Variability in Marine Atmospheric Mercury Isotope Signatures and Environmental Drivers

Zhengcheng Song[1,2,3*], Xin Miao[2], Congyuan Li[4], Yujuan Wang[2], Shaojian Huang[2], Peng Zhang[2], Kaihui Tang[1,5], Tengfei Yuan[6], Yanxu Zhang[6*]

[1]State Key Laboratory of Environmental Geochemistry, Institute of Geochemistry, Chinese Academy of Sciences, Guiyang, 550081, China

[2]School of Atmospheric Sciences, Nanjing University, Nanjing, Jiangsu, 210023, China

[3]Frontiers Science Center for Critical Earth Material Cycling, Nanjing University, Nanjing, Jiangsu, 210023, China

[4]College of Meteorology and Oceanography, National University of Defense Technology, Changsha, 410073, China

[5]University of Chinese Academy of Sciences, Beijing, 100049, China

[6]Department of Earth and Environmental Sciences, Tulane University, New Orleans, LA, 70118, United Sates

*Corresponding Authors: ZS: songzc@nju.edu.cn and YZ: yzhang127@tulane.edu

This file includes:

Pages: 16

Table S1 to S2

Figure S1 to S10

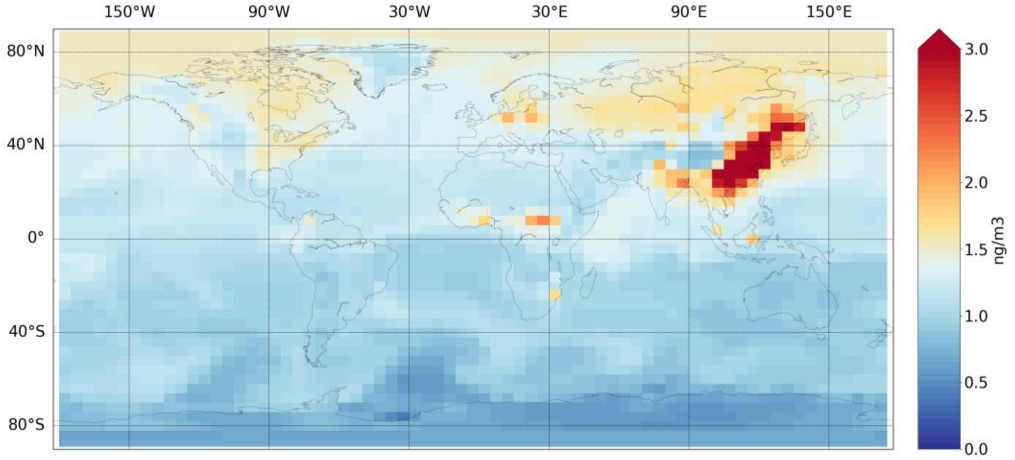

Figure S1. Atmospheric Hg concentrations in the surface air under the initial atmospheric conditions.

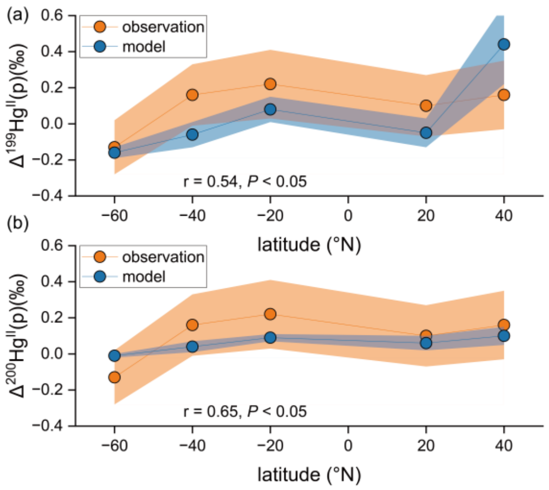

Figure S2. Comparison of observed and modeled latitudinal MIF signatures in $Hg^{II}(p)$. (a) $\Delta^{199}Hg$ signatures in $Hg^{II}(p)$. (b) $\Delta^{200}Hg$ signatures in $Hg^{II}(p)$. Observation sites are located between 60°S and 40°N, as compiled from the referenced studies.[1,2]

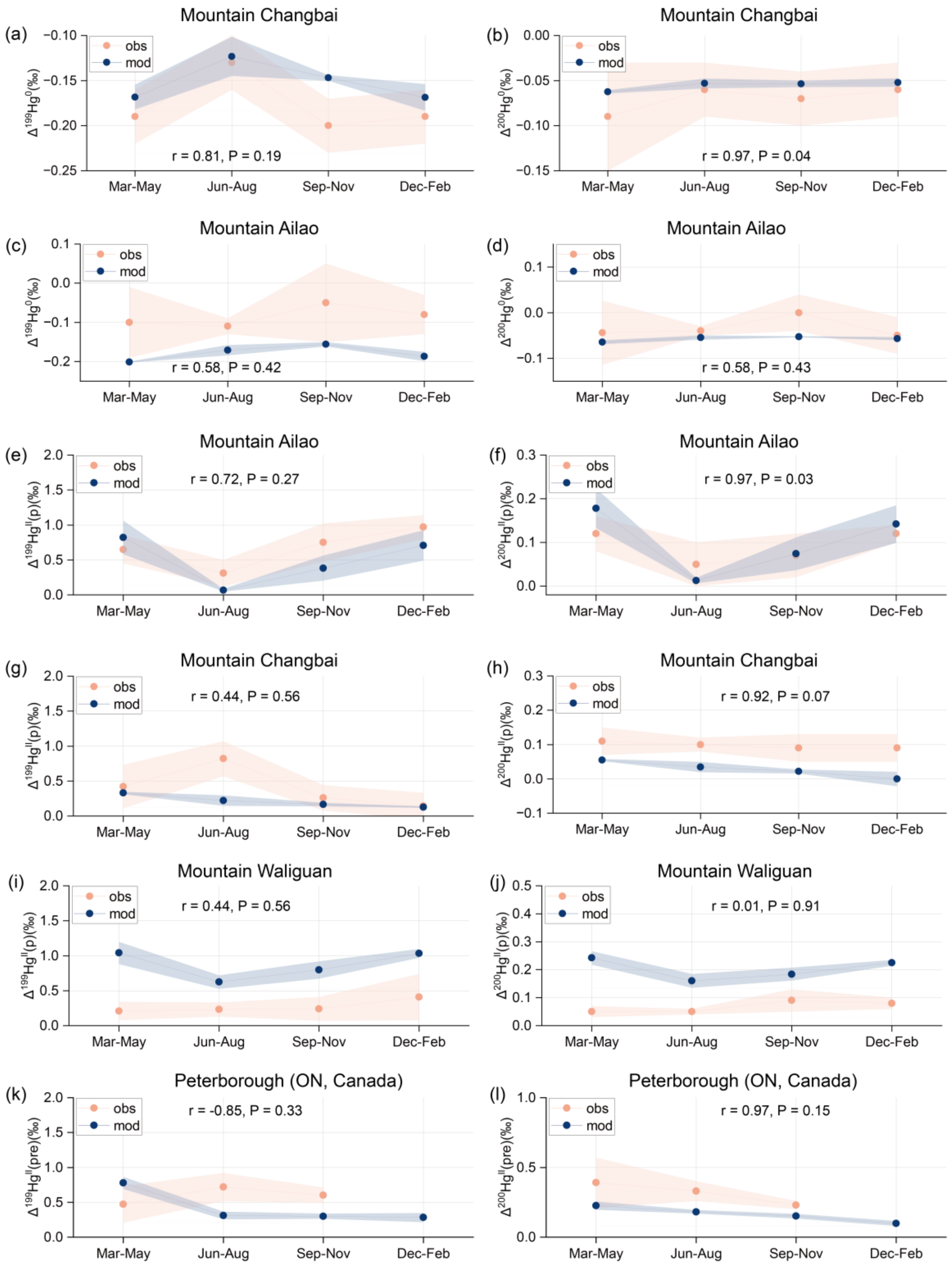

Figure S3. Comparison of observed and modeled seasonal variation of MIF signatures of atmospheric Hg. (a)-(b) Seasonality of $\Delta^{199}Hg^{0}$ and $\Delta^{200}Hg^{0}$ signatures at mountain Changbai region.[3] (c)-(d) Seasonality of $\Delta^{199}Hg^{0}$ and $\Delta^{200}Hg^{0}$ signatures at mountain Ailao region.[3] (e)-(f) Seasonality of $\Delta^{199}Hg^{II}(p)$ and $\Delta^{200}Hg^{II}(p)$ signatures at mountain Ailao region.[4] (g)-(h) Seasonality of $\Delta^{199}Hg^{II}(p)$ and $\Delta^{200}Hg^{II}(p)$ signatures at mountain Changbai region.[4] (i)-(j) Seasonality of $\Delta^{199}Hg^{II}(p)$ and $\Delta^{200}Hg^{II}(p)$ signatures at mountain Waliguan region.[4] (k)-(l) Seasonality of $\Delta^{199}Hg^{II}(pre)$ and $\Delta^{200}Hg^{II}(pre)$ signatures at Peterborough, ON, Canada.[5]

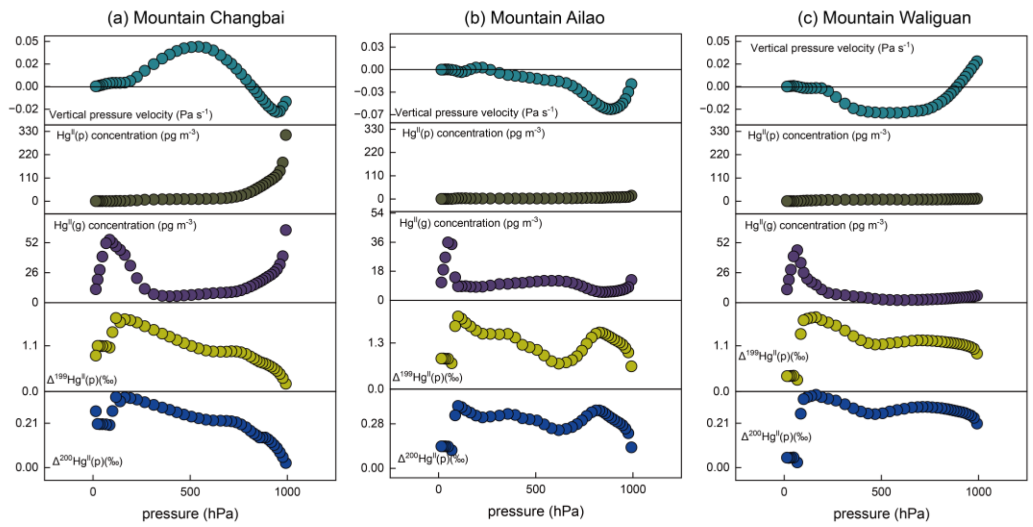

Figure S4. Vertical profiles of modeled annual-mean pressure velocity, Hg^{II} concentrations, and MIF signatures of Hg^{II}(p) at (a) Mountain Changbai, (b) Mountain Ailao, and (c) Mountain Waliguan. Positive pressure velocity indicates subsidence (downward motion), whereas negative values indicate upward atmospheric motion.

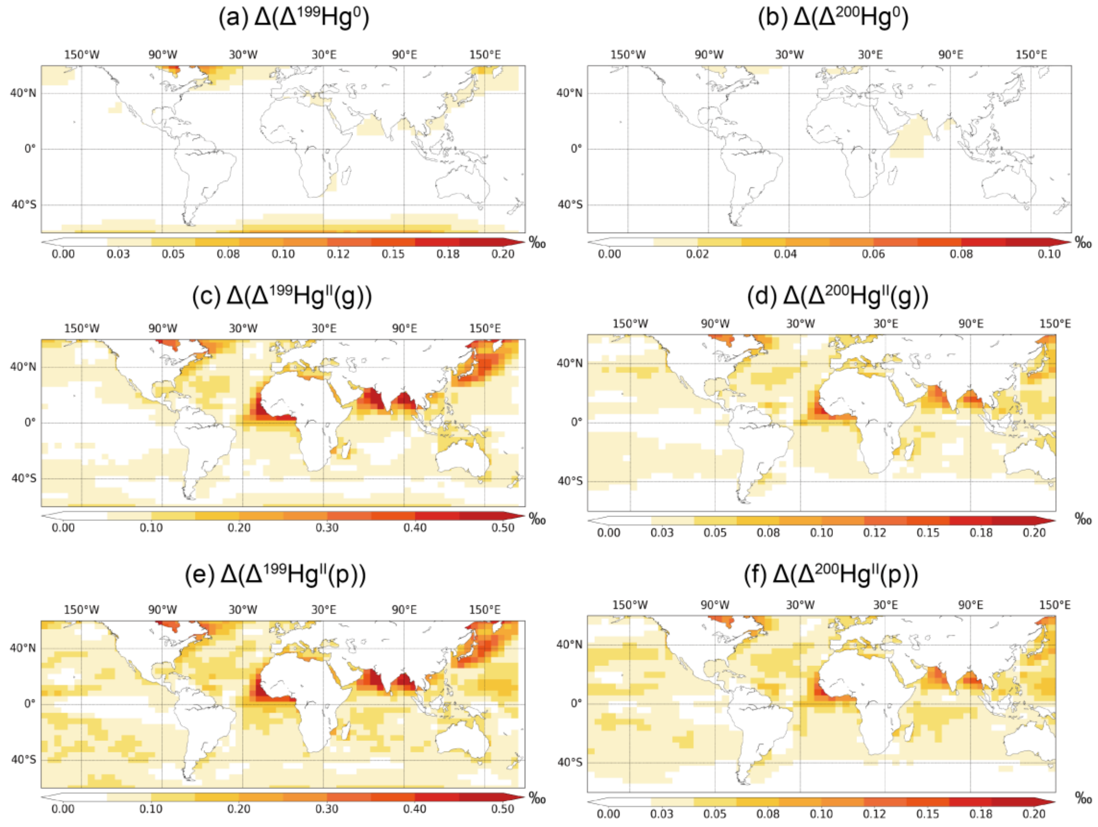

Figure S5. Standard deviations (Δ) of monthly MIF signatures in the marine boundary layer. The Standard deviations are calculated by 12 months simulated MIF signatures in the isotope model. (a) Δ^{199}Hg values changes in Hg^{0}. (b) Δ^{200}Hg values changes in Hg^{0}. (c) Δ^{199}Hg values changes in Hg^{II}(g). (d) Δ^{200}Hg values changes in Hg^{II}(g). (e) Δ^{199}Hg values changes in Hg^{II}(p). (f) Δ^{200}Hg values changes in Hg^{II}(p).

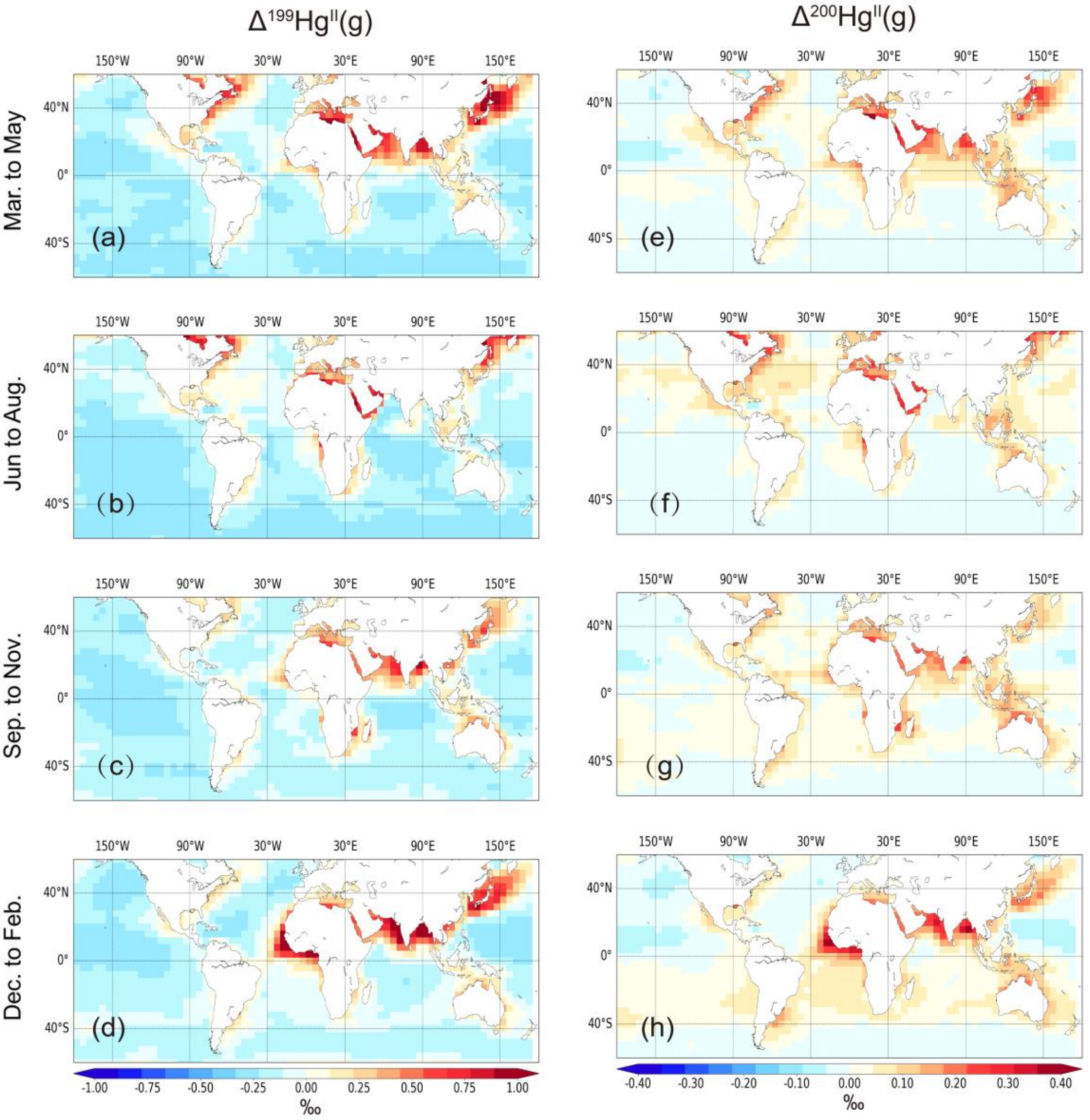

Figure S6. Seasonal variation of MIF signatures of $Hg^{II}(g)$ in the MBL of non-polar regions (60°S to 60°N). The seasons is distinguished according to North Hemisphere, which comprises of spring (March to May), summer (Jun to August), autumn (September to November), winter (December to February). (a)-(d) $\Delta^{199}Hg^{II}(g)$ signatures in the four seasons, respectively. (e)-(h) $\Delta^{200}Hg^{II}(g)$ signatures in the four seasons, respectively.

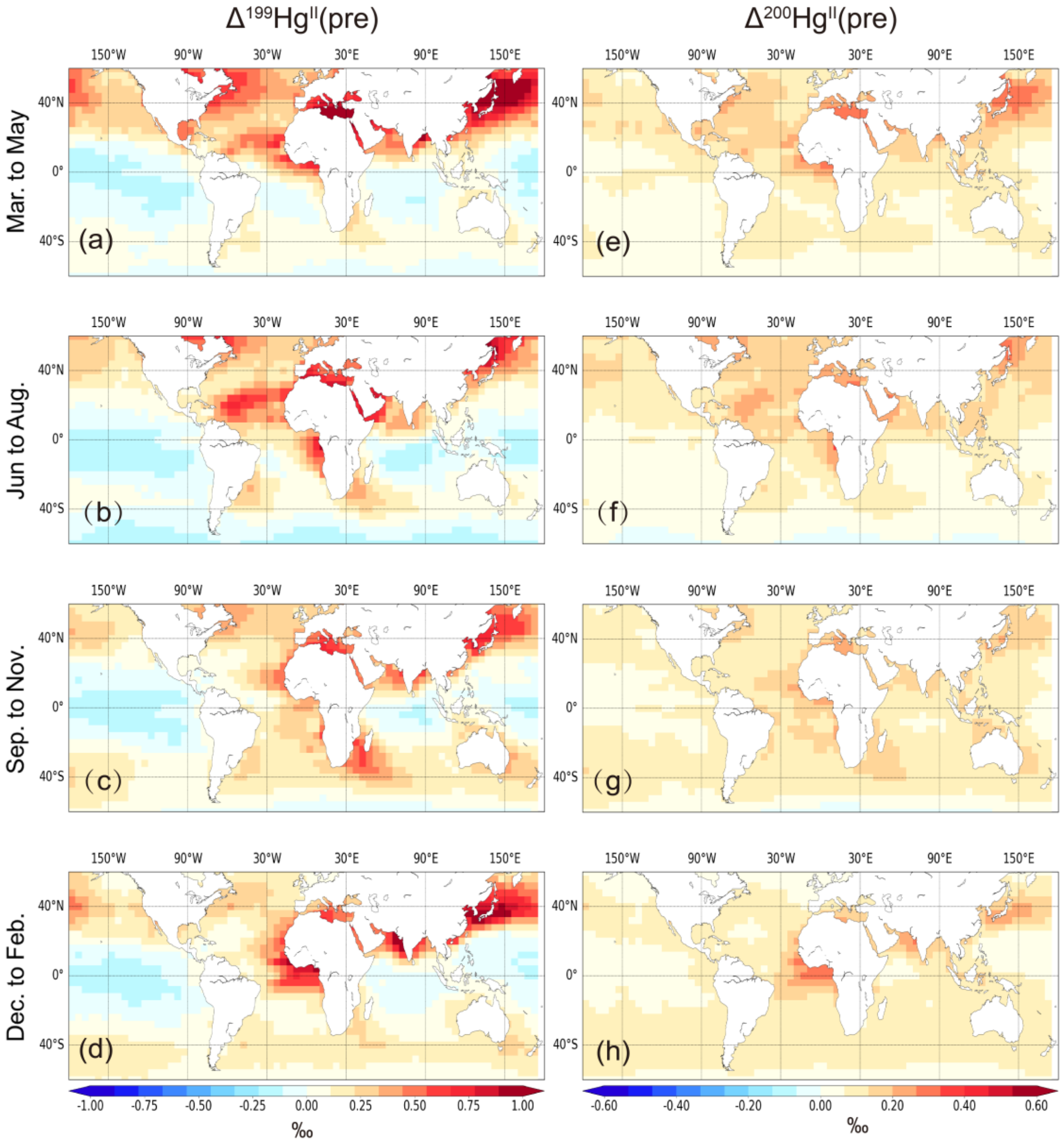

Figure S7. Seasonal variation of MIF signatures of Hg^{II}(pre) in the MBL of non-polar regions (60°S to 60°N). The seasons is distinguished according to North Hemisphere, which comprises of spring (March to May), summer (Jun to August), autumn (September to November), winter (December to February). (a)-(d) $\Delta^{199}Hg^{II}$(pre) signatures in the four seasons, respectively. (e)-(h) $\Delta^{200}Hg^{II}$(pre) signatures in the four seasons, respectively.

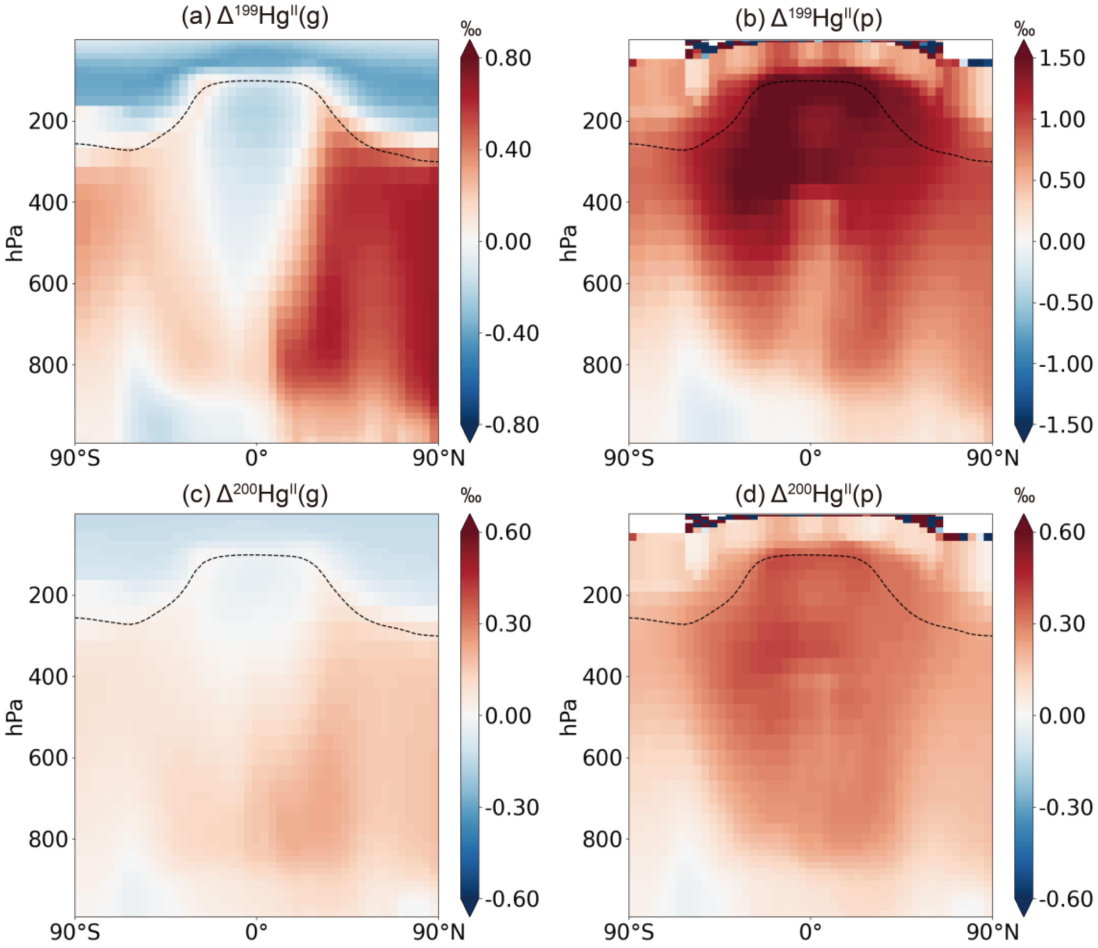

Figure S8. Annual zonal mean of (a) $\Delta^{199}Hg^{II}(g)$, (b) $\Delta^{199}Hg^{II}(g)$, (c) $\Delta^{200}Hg^{II}(g)$, and (d) $\Delta^{200}Hg^{II}(p)$ in global atmosphere.

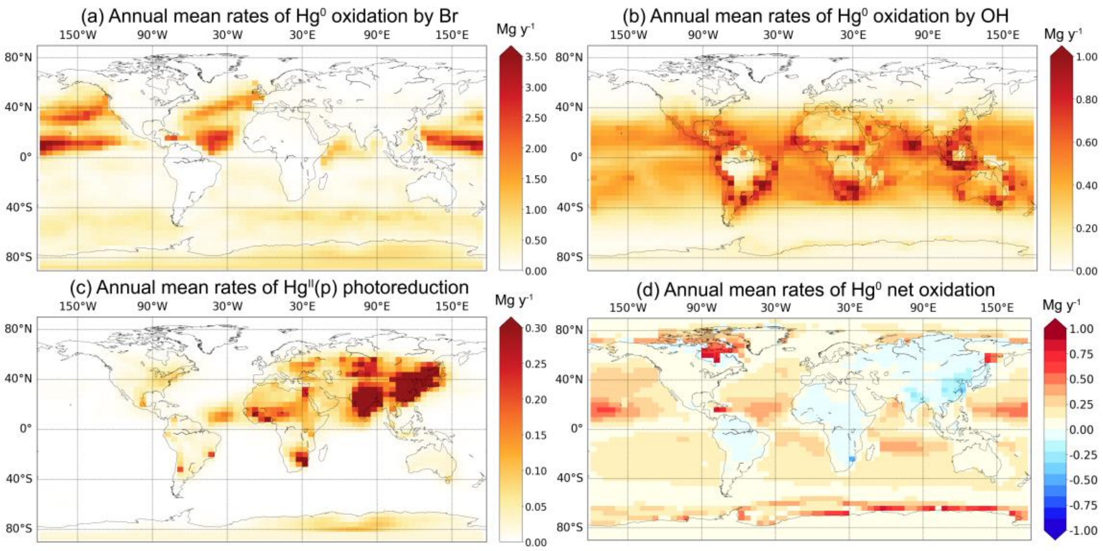

Figure S9. Annual mean chemistry rates of (a) Hg^0 oxidation by Br, (b) Hg^0 oxidation by OH, (c) reduction of Hg^{II}(p), and (d) net oxidation of Hg^0 in the surface atmosphere.

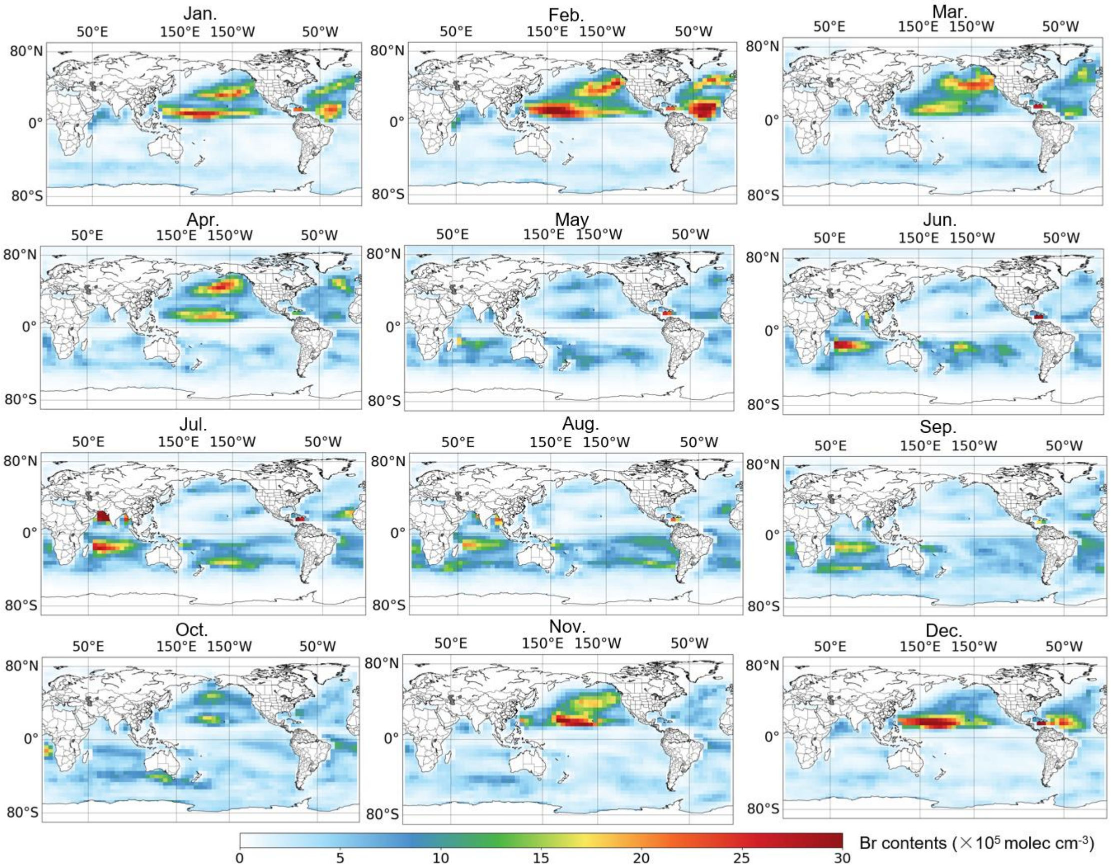

Figure S10. Monthly mean of Br contents in global marine boundary layer.

Table S1 Isotope signatures of Hg end-members emitted from emission sources.

Sources	Δ^{199}Hg(‰)	SD	Δ^{200}Hg(‰)	SD	References
Soil (850 Mg yr^{-1})[a]	0.25	0.14	−0.04	0.02	6, 7
Snow (300 Mg yr^{-1})[b]	—	—	0.00	0.00	8
Ocean (4800Mg yr^{-1})[c]	0.08	0.18	0.04	0.05	9
Geogenic (300 Mg yr^{-1})[d]	0.05	0.06	0.00	0.02	10
Biomass buring (300 Mg yr^{-1})[e]	−0.21	0.02	−0.04	0.02	11, 12
Anthropogenic emissions (2200 Mg yr^{-1})[f]	−0.07	0.11	0.00	0.03	10

The emission fluxes are from Shah et al [13]. [a]Mercury stable isotope signatures of soil is sysithized in Sun et al.[7], here we use the average values from organic surface soils and mineral soils. [b]Odd-MIF signatures are not intergrated for snow, as Hg in snow is inheret from the atmosphere in the model. While we imply MIF effect for snow emission processes following the Chamber experiments[8, 14]. Even-MIF signatures in snow-released Hg^0 are assumed zero according observations from Sherman et al. [8]. [c]Oceaic Hg isotope signatures is utilized from the total Hg in the ocean as reported by Jiskra et al.[9]. We fitted a E^{199}Hg of −0.29‰ from Zhang et al.[15], with the Δ^{199}Hg in oceanic released Hg^0 of about −0.2‰. [d]Geogenic source is cited from Sun et al.[7], which reports Hg isotope observations on bulk volcanic emissions. [e]Mercury isotope signatures of biomass buring source are sysithized in Fu et al.[12] and Kruz et al.[11], here we utilize average values from these studies. [f]Sun et al.[10] reportes Hg isotopic signatures for various anthropogenic sources, and Song et al[16] establishes Hg isotopic inventories for global anthropogenic sources.

Table S2 The min and max monthly mean MIF signatures modeled by sensitive simulations.

	original fractionation factors					
Indian ocean	Hg^0 deposition		Hg^{II} dry deposition		Hg^{II} wet deposition	
	$\Delta^{199}Hg$(‰)	$\Delta^{200}Hg$(‰)	$\Delta^{199}Hg$(‰)	$\Delta^{200}Hg$(‰)	$\Delta^{199}Hg$(‰)	$\Delta^{200}Hg$(‰)
min	-0.32	-0.07	-0.25	-0.02	-0.11	0.04
max	-0.30	-0.05	-0.12	0.04	0.04	0.09
North Pacific Ocean						
min	-0.35	-0.08	-0.23	-0.04	0.05	0.05
max	-0.30	-0.06	-0.12	0.02	0.31	0.15
South Pacific Ocean						
min	-0.32	-0.06	-0.25	-0.03	-0.08	0.04
max	-0.29	-0.05	-0.13	0.03	0.03	0.09
North Atlantic						
min	-0.35	-0.09	-0.15	-0.01	0.16	0.08
max	-0.31	-0.07	0.03	0.06	0.45	0.18
	**fractionation factors in oxidation reactions x2**					
Indian ocean	Hg^0 deposition		Hg^{II} dry deposition		Hg^{II} wet deposition	
	$\Delta^{199}Hg$(‰)	$\Delta^{200}Hg$(‰)	$\Delta^{199}Hg$(‰)	$\Delta^{200}Hg$(‰)	$\Delta^{199}Hg$(‰)	$\Delta^{200}Hg$(‰)
min	-0.07	-0.10	-0.45	-0.04	-0.28	0.05
max	-0.04	-0.08	-0.33	0.07	-0.11	0.15
North Pacific Ocean						
min	-0.10	-0.11	-0.47	-0.06	-0.14	0.06
max	-0.08	-0.09	-0.33	0.05	0.19	0.20
South Pacific Ocean						
min	-0.06	-0.10	-0.46	-0.04	-0.25	0.05
max	-0.03	-0.08	-0.31	0.06	-0.11	0.13
North Atlantic						
min	-0.11	-0.12	-0.36	-0.03	0.00	0.09
max	-0.09	-0.10	-0.16	0.09	0.38	0.24
	**fractionation factors in Hg^{II}(p) photoreduction x2**					
Indian ocean	Hg^0 deposition		Hg^{II} dry deposition		Hg^{II} wet deposition	
	$\Delta^{199}Hg$(‰)	$\Delta^{200}Hg$(‰)	$\Delta^{199}Hg$(‰)	$\Delta^{200}Hg$(‰)	$\Delta^{199}Hg$(‰)	$\Delta^{200}Hg$(‰)
min	-0.49	-0.12	-0.54	-0.06	-0.22	0.03
max	-0.42	-0.09	-0.29	0.03	0.13	0.11
North Pacific Ocean						
min	-0.49	-0.12	-0.54	-0.06	-0.22	0.03

max	-0.42	-0.09	-0.29	0.03	0.13	0.11
South Pacific Ocean						
min	-0.46	-0.11	-0.55	-0.06	-0.14	0.04
max	-0.41	-0.09	-0.29	0.02	0.11	0.11
North Atlantic						
min	-0.62	-0.15	-0.34	-0.03	0.35	0.13
max	-0.51	-0.12	0.05	0.08	1.08	0.29

References

1. Au Yang, D.; Chen, J.; Zheng, W.; Zhang, Y.; Shi, G.; Sonke, J. E.; Cartigny, P.; Cai, H.; Yuan, W.; Liu, L.; Gai, P.; Liu, C., South-hemispheric marine aerosol Hg and S isotope compositions reveal different oxidation pathways; National Science Open, 2022: 20220014. 10.1051/nso/2021001

2. Qiu, Y.; Gai, P. X.; Yue, F. G.; Zhang, Y. Y.; He, P. Z.; Kang, H.; Yu, X. W.; Lam, P. K. S.; Chen, J. B.; Xie, Z. Q., Stable Mercury Isotopes Revealing Photochemical Processes in the Marine Boundary Layer; J Geophys Res-Atmos, 2021, 126: e2021JD034630. ARTN e2021JD034630 10.1029/2021JD034630

3. Fu, X.; Zhang, H.; Liu, C.; Zhang, H.; Lin, C. J.; Feng, X., Significant Seasonal Variations in Isotopic Composition of Atmospheric Total Gaseous Mercury at Forest Sites in China Caused by Vegetation and Mercury Sources; Environmental science & technology, 2019, 53: 13748-13756. 10.1021/acs.est.9b05016

4. Fu, X.; Zhang, H.; Feng, X.; Tan, Q.; Ming, L.; Liu, C.; Zhang, L., Domestic and Transboundary Sources of Atmospheric Particulate Bound Mercury in Remote Areas of China: Evidence from Mercury Isotopes; Environmental science & technology, 2019, 53: 1947-1957. 10.1021/acs.est.8b06736

5. Chen, J. B.; Hintelmann, H.; Feng, X. B.; Dimock, B., Unusual fractionation of both odd and even mercury isotopes in precipitation from Peterborough, ON, Canada; Geochimica Et Cosmochimica Acta, 2012, 90: 33-46. 10.1016/j.gca.2012.05.005

6. Wang, X.; Yuan, W.; Lin, C. J.; Zhang, L.; Zhang, H.; Feng, X., Climate and Vegetation As Primary Drivers for Global Mercury Storage in Surface Soil; Environmental science & technology, 2019, 53: 10665-10675. 10.1021/acs.est.9b02386

7. Sun, R.; Jiskra, M.; Amos, H. M.; Zhang, Y.; Sunderland, E. M.; Sonke, J. E., Modelling the mercury stable isotope distribution of Earth surface reservoirs: Implications for global Hg cycling; Geochimica et Cosmochimica Acta, 2019, 246: 156-173. 10.1016/j.gca.2018.11.036

8. Sherman, L. S.; Blum, J. D.; Johnson, K. P.; Keeler, G. J.; Barres, J. A.; Douglas, T. A., Mass-independent fractionation of mercury isotopes in Arctic snow driven by sunlight; Nat Geosci, 2010, 3: 173-177. 10.1038/Ngeo758

9. Jiskra, M.; Heimbürger-Boavida, L.-E.; Desgranges, M.-M.; Petrova, M. V.; Dufour, A.; Ferreira-Araujo, B.; Masbou, J.; Chmeleff, J.; Thyssen, M.; Point, D.; Sonke, J. E., Mercury stable isotopes constrain atmospheric sources to the ocean; Nature, 2021, 597: 678-682. 10.1038/s41586-021-03859-8

10. Sun, R. Y.; Streets, D. G.; Horowitz, H. M.; Amos, H. M.; Liu, G. J.; Perrot, V.; Toutain, J. P.; Hintelmann, H.; Sunderland, E. M.; Sonke, J. E., Historical (1850-2010) mercury stable isotope inventory from anthropogenic sources to the atmosphere; Elementa-Sci Anthrop, 2016, 4: 1-15. ARTN 000091
10.12952/journal.elementa.000091

11. Kurz, A. Y.; Blum, J. D.; Gratz, L. E.; Jaffe, D. A., Contrasting Controls on the Diel Isotopic Variation of Hg(0) at Two High Elevation Sites in the Western United States; Environmental science & technology, 2020, 54: 10502-10513. 10.1021/acs.est.0c01918

12. Fu, X.; Yang, X.; Tan, Q.; Ming, L.; Lin, T.; Lin, C.-J.; Li, X.; Feng, X., Isotopic Composition of Gaseous Elemental Mercury in the Marine Boundary Layer of East China Sea; Journal of Geophysical Research: Atmospheres, 2018, 123: 7656-7669. 10.1029/2018jd028671

13. Shah, V.; Jacob, D. J.; Thackray, C. P.; Wang, X.; Sunderland, E. M.; Dibble, T. S.; Saiz-Lopez, A.; Černušák, I.; Kellö, V.; Castro, P. J.; Wu, R.; Wang, C., Improved Mechanistic Model of the Atmospheric Redox Chemistry of Mercury; Environmental science & technology, 2021, 55: 14445–

14456. 10.1021/acs.est.1c03160
14. Douglas, T. A.; Blum, J. D., Mercury Isotopes Reveal Atmospheric Gaseous Mercury Deposition Directly to the Arctic Coastal Snowpack; Environmental Science & Technology Letters, 2019, 6: 235-242. 10.1021/acs.estlett.9b00131
15. Zhang, H.; Fu, X.; Wu, X.; Deng, Q.; Tang, K.; Zhang, L.; Sommar, J.; Sun, G.; Feng, X., Using Mercury Stable Isotopes to Quantify Bidirectional Water–Atmosphere Hg(0) Exchange Fluxes and Explore Controlling Factors; Environmental science & technology, 2023, 57: 10673-10685. 10.1021/acs.est.3c01273
16. Song, Z.; Sun, R.; Zhang, Y., Modeling mercury isotopic fractionation in the atmosphere; Environmental pollution, 2022: 119588. 10.1016/j.envpol.2022.119588
